# 132 Changes in Antibiotic Consumption Following Reimplementation of an Antimicrobial Stewardship Program after Temporary Suspension

**DOI:** 10.1017/ash.2026.10544

**Published:** 2026-06-23

**Authors:** Casey Morrell, Melphine Harriott, Christopher Evans, Christopher Wilson, Simone Godwin

**Affiliations:** 1 Tennessee Department of Health; 2 TN Department of Health HAI/AR Program; 3 TN Department of Health

## Abstract

**Background:** As part of the Centers for Disease Control and Prevention’s (CDC’s) Emerging Infections Program (EIP), the Tennessee Department of Health (TDH) conducts surveillance on Clostridioides difficile infection (CDI) in a designated catchment area. As part of ongoing surveillance, select TDH staff have access to Centers for Medicare and Medicaid Services (CMS) patient-level data under a CDC-CMS data use agreement. The aim of this study was to assess the alignment between the most prevalent chronic conditions among CDI cases reported through EIP and those reported through CMS. **Methods:** We matched patients who were 65 years and older from 2022 EIP data with Medicare beneficiaries on date of birth, sex, county of residence, and ZIP code, and retained unique matches for our study. Comorbidities present in both the CMS 30 CCW Chronic Conditions list and in the CDI case report form were included. The most prevalent chronic conditions in each dataset were identified. We employed Cohen's Kappa to assess inter-rater reliability between chronic conditions reported through EIP chart abstractions and those reported via CMS claim submissions. A Cohen’s Kappa greater than 0.4 indicates moderate or greater inter-rater reliability. Analysis was conducted using SAS Studio. **Results:** Of the 431 EIP CDI patients in 2022, 196 patients were 65 years or older and eligible for inclusion. 130 of these patients matched uniquely to a Medicare beneficiary ID on the demographics listed above (66.3%). The most prevalent chronic conditions identified among CDI patients were cardiovascular disease (CVD) (n=31), diabetes mellitus (n=26), chronic kidney disease (CKD) (n=21), and chronic pulmonary disease (CPD) (n=14). Diabetes mellitus diagnoses between the two datasets had the highest concordance with a Cohen’s Kappa of 0.5226 (p<0.0001), followed by CKD at 0.3984 (p<0.0001), CPD at 0.2313 (p=0.0081), and CVD at 0.1371 (p=0.1162). **Conclusions:** Only diabetes mellitus diagnoses showed moderate concordance between EIP and CMS datasets. EIP processes utilize chart review to generate surveillance data, which is person-power intensive. This limited study indicates that comorbidity data for CDI patients may not be completely captured if surveillance was conducted through secondary analyses using CMS data rather than complete chart review by trained public health staff.

